# Short- versus long-course antibiotic therapy in mechanically ventilated sepsis patients with pneumonia: a real-world cohort analysis

**DOI:** 10.1016/j.clinsp.2026.100932

**Published:** 2026-04-11

**Authors:** Bin Wang, Shuiqing Gui, Jiang Mei, Zhiye Zou

**Affiliations:** a Department of Ultrasound, Longgang Central Hospital of Shenzhen, Shenzhen, China; b Department of Critical Care Medicine, Shenzhen Second People’s Hospital & First Affiliated Hospital of Shenzhen University, Shenzhen, China

**Keywords:** Pneumonia, Sepsis, Antibiotic, Duration

## Abstract

**Objective:**

To compare outcomes of short-course versus longer-course antibiotic regimens in mechanically ventilated adult patients with sepsis due to pneumonia.

**Methods:**

The authors performed a retrospective cohort study using the MIMIC-IV database (2008–2022), identifying 2590 adults meeting Sepsis-3 criteria with a primary diagnosis of pneumonia requiring invasive mechanical ventilation. Patients were stratified by antibiotic duration (short: 2‒7 days; long: 8‒14 days). Demographics, comorbidities, severity (SOFA score, PaO_2_/FiO_2_ ratio), and microbiology were extracted. The primary outcome was 90-day all-cause mortality. The authors used Kaplan-Meier analysis, multivariable Cox proportional hazards models, and subgroup analyses to assess the effect of treatment duration.

**Results:**

The study found that overall 90-day mortality did not differ significantly (short-course ∼41.3% vs. long-course ∼41.2%; *p* = 0.98). Subgroup analyses indicated that in patients > 65-years, those with severe hypoxemia (low PaO_2_/FiO_2_), on RRT, or infected with Staphylococcus aureus or Pseudomonas aeruginosa, shorter therapy was associated with higher mortality rates (*p* < 0.05). Conversely, younger or less severely ill patients showed no significant effect of duration. These findings may reflect residual confounding.

**Conclusions:**

In this observational cohort, shorter antibiotic courses were not associated with increased 90‑day mortality after adjustment. Given potential confounding and limited recurrence data, subgroup signals are hypothesis‑generating and require confirmation in randomized trials.

## Highlights

• Comparable 90-day survival: In 2590 sepsis patients, short (2–7 days) and long (8–14 days) antibiotic courses showed similar outcomes (∼41%, *p* = 0.98).
• High-risk subgroups identified: Extended therapy reduced mortality in patients > 65-years, severe hypoxemia, renal replacement therapy, or infections with *Staphylococcus aureus* or *Pseudomonas aeruginosa*.
• Supports antimicrobial stewardship: Short-course therapy is safe in lower-risk patients, aiding in efforts to minimize antibiotic overuse.
• Advocates for personalized treatment: Findings suggest antibiotic duration should be tailored based on patient factors, pathogen, and severity.

## Introduction

Sepsis causes massive global morbidity and mortality.^1^ Among infection sites, pneumonia is the most common source of sepsis and is associated with higher mortality than other foci.^2^ In mechanically ventilated patients with sepsis from pneumonia, early recognition and prompt initiation of appropriate broad-spectrum antibiotics are cornerstones of management and are linked to improved survival.^3^ However, the debate over the duration of antibiotic treatment remains intense.^4^

While traditionally prolonged courses have been used, there is increasing evidence that shorter regimens can be as effective as longer ones in many infections.^5^ Recent trials and meta-analyses have demonstrated that abbreviated courses can maintain efficacy; for instance, pediatric studies in Community-Acquired Pneumonia (CAP) have shown that 3–5 day courses are non-inferior to longer regimens in healthy children.^6^ Similarly, in adult CAP, shorter courses (5–7 days) achieved outcomes comparable to 8–10 day courses.^7^ Critically, the impact of extended versus abbreviated antibiotic courses on outcomes in mechanically ventilated pneumonia with sepsis has not been established.

The optimal duration of antibiotic therapy in mechanically ventilated patients with sepsis from pneumonia is uncertain.^8^ To address this gap, the authors investigated outcomes in ventilated patients with sepsis from pneumonia treated with short- and long-course antibiotics. The primary endpoint was 90-day mortality. Additionally, the authors examined whether the effect of antibiotic duration differed among various clinical subgroups, such as patient baseline characteristics, organ dysfunction, and pathogen type. This study aims to inform evidence-based antibiotic stewardship for critically ill pneumonia patients.

## Materials and methods

### Methods

The authors conducted a retrospective cohort study using the publicly available Medical Information Mart for Intensive Care IV (MIMIC-IV) database (2008–2022)^9^ of adult ICU patients. Population: All ICU admissions of patients aged ≥ 18-years with pneumonia complicated by sepsis, defined by Sepsis-3 criteria (documented or suspected infection plus an acute increase in Sequential Organ Failure Assessment (SOFA) score ≥ 2), were screened.^10^ Only patients requiring invasive mechanical ventilation within 48 hours of admission were included. Exclusion criteria were: Aged < 18 and age ≥ 95; Stayed in the ICU less than 3-days or more than 100 days; Died in the ICU less than 3 days; Mechanical ventilation in the ICU less than 2 days; Sepsis was initiated 12 h before or 24 h after ICU admission; The initiation time of anti-infection was more than 24 hours; The duration of anti-infection in ICU was less than 2-days and more than 14-days; Had multiple admissions other than the first admission. After screening, 2590 patients met the inclusion criteria. The report complies with the recommendations of STROBE (Strengthening the Reporting of Observational Studies in Epidemiology).

### Data and definitions

Demographics, vital signs, laboratory values, and clinical scores were extracted. Antibiotic therapy was identified from medication records. Antibiotics were categorized by class (β-lactams, macrolides, fluoroquinolones, etc.), and duration was calculated as the total consecutive days of administration. The authors defined a short-course regimen as 2‒7 days of initial therapy, and a long-course regimen as 8‒14 days, consistent with clinical experience, prior studies and guidelines.^11^^,^^12^ Time to antibiotic initiation was defined as the duration from ICU admission, calculated as the time of antibiotic administration minus the time of ICU admission. Mechanical ventilation was defined as any episode of invasive ventilation lasting > 24 h. Pathogens were identified from culture results (respiratory and blood).

### Statistical analysis

Baseline characteristics were compared between short- and long-course groups using chi-square or Fisher’s exact tests for categorical variables and Student’s *t*-test or Mann-Whitney *U* test for continuous variables, as appropriate.

The primary outcome was 90-day all-cause mortality from the day of ICU admission. Survival was analyzed using Kaplan-Meier curves with log-rank tests. Multivariable Cox proportional hazards regression was used to estimate Hazard Ratios (HR) for 90-day mortality associated with antibiotic duration, adjusting for age, sex, Charlson comorbidity index, initial SOFA score, PaO_2_/FiO_2_ ratio, vasopressor use, RRT, and pathogen type. The authors also performed Propensity Score Matching (PSM), incorporating all variables from Table 1. A caliper width of 0.05 was employed for one-to-one nearest neighbor matching without replacement. Post-matching balance was assessed using Standardized Mean Differences (SMDs), with a threshold of > 0.1 indicating an imbalance between groups (Fig. S1). After matching, 1050 patients were included in each group for further analysis.

**Table 1:** Baseline characteristics and clinical variables.

Variables^a^	Short-course therapy (*n* = 1355)	Long-course therapy (*n* = 1235)	p-value
Age (years)	66.7 (56.5, 77.5)	64.6 (52.8, 74.7)	<0.001
Gender (male)	785 (57.9)	724 (58.6)	0.72
Weight (kg)	78.8 (65.0, 95.1)	80.0 (67.0, 98.2)	0.048
Emergency admission	684 (50.5)	673 (54.5)	0.041
White race	821 (60.6)	774 (62.7)	0.28
Medicare insurance	789 (58.2)	652 (52.8)	0.005
History of disease			
Hypertension	924 (68.2)	773 (62.6)	0.003
Myocardial infarct	276 (20.4)	196 (15.9)	0.003
Diabetes Mellitus	453 (33.4)	364 (29.5)	0.030
Chronic pulmonary disease	519 (38.3)	398 (32.2)	0.001
Chronic kidney disease	343 (25.3)	277 (22.4)	0.086
Malignant cancer	190 (14.0)	166 (13.4)	0.67
Vital signs on day-1			
Maximum heart rate (bpm)	107.0 (92.0, 123.0)	113.0 (98.0, 127.0)	<0.001
Maximum body temperature( °C)	37.6 (37.1, 38.3)	37.7 (37.2, 38.4)	0.002
Minimum PaO_2_/FiO_2_ ratio	158.9 (107.0, 237.5)	148.0 (90.0, 220.0)	<0.001
Minimum MAP (mmHg)	56.0 (49.0, 62.0)	56.0 (49.0, 62.0)	0.59
Scoring system on day-1			
Maximum OASIS score	39.0 (34.0, 44.0)	39.0 (34.0, 45.0)	0.48
Maximum APS III score	57.0 (43.0, 73.0)	61.0 (47.0, 76.0)	<0.001
Maximum SOFA score	8.0 (5.0, 11.0)	8.0 (6.0, 11.0)	0.028
Renal replacement therapy on day-1	59 (4.4)	64 (5.2)	0.32
Norepinephrine use on day-1	546 (40.3)	556 (45.0)	0.015
Time to antibiotic initiation (hours)	3.9 (2.0, 10.9)	3.6 (1.8, 11.3)	0.40
Time from admission to sepsis (hours)	1.4 (0.8, 3.1)	1.5 (0.8, 3.5)	0.26

bpm, Beat per minute or breaths per minute; MAP, Mean Blood Pressure; OASIS, Oxford Acute Severity of Illness; SAP III, Simplified Acute Physiology score III; SOFA, Sequential Organ Failure Assessment.

a Because all continuous variables were not normally distributed, the continuous variables were expressed as median (IQR). Categorical variables were expressed as n (%).

The authors tested interaction terms and performed subgroup analyses stratified by age (> 65 vs. ≤ 65), baseline PaO_2_/FiO_2_ (< 150 vs. ≥ 150), use of renal replacement therapy, and presence of Staphylococcus aureus or Pseudomonas aeruginosa infection.

A two-sided *p* < 0.05 was considered statistically significant. Statistical analyses were performed using R (version 4.3.1) and Stata (version 18).

Ethics: The use of MIMIC-IV data was approved in accordance with the institutional review board protocol for the database. Access to the database is granted to individuals who successfully complete the Collaborative Institutional Training Initiative test (author ZYZ’s certification number: 59,729,494). All data are fully de-identified, and patient consent requirements were waived as per policy.

## Results

### Baseline characteristics

**Fig. 1:**
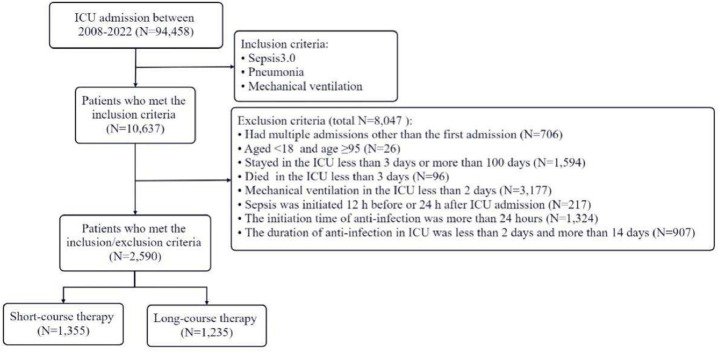
Flowchart of patient selection.

A total of 10,637 septic patients were initially screened, of whom 2590 met all predefined inclusion and exclusion criteria for this analysis (Fig. 1). All patients were diagnosed with sepsis secondary to pneumonia and required mechanical ventilation during their ICU stay. Empiric antibiotic therapy was initiated within 24 hours of ICU admission in all cases. Among the included patients, 1355 (52.3%) received short-course antibiotic therapy, with a median duration of 4.6-days (Interquartile Range [IQR]: 3.6–5.8), whereas 1235 (47.7%) received long-course therapy, with a median duration of 9.7 days (IQR: 8.2–11.8).

Compared to those in the long-course group, patients in the short-course group were older (mean age 66.7 vs. 64.6 years, *p* < 0.001) and had a higher prevalence of hypertension (68.2% vs. 62.6%, *p* < 0.001) and chronic pulmonary disease (38.3% vs. 32.2%, *p* = 0.001). In contrast, the long-course group demonstrated greater illness severity, as indicated by higher maximum APS III scores (61.0 vs. 57.0, *p* < 0.001) and slightly elevated maximum SOFA scores (median 8.0 in both groups, *p* = 0.028). Furthermore, vasopressor support with norepinephrine was more frequently required in the long-course group (45.0% vs. 40.3%, *p* = 0.015). No statistically significant differences were observed between groups regarding renal replacement therapy on ICU day-1, time from hospital admission to sepsis onset, or time to antibiotic initiation (Table 1).

Microbiological profiles revealed no significant difference in the proportion of Multidrug-Resistant (MDR) pulmonary pathogens between groups (15.1% vs. 14.5%, *p* = 0.65) (Table 2). However, several organisms were more frequently isolated in the long-course group, including *Staphylococcus aureus* (21.3% vs. 15.4%, *p* < 0.001), *Escherichia coli* (7.9% vs. 3.8%, *p* < 0.001), and fungal pathogens (47.5% vs. 34.7%, *p* < 0.001). Co-detection of respiratory viruses was also higher in the long-course group (7.4% vs. 4.8%, *p* = 0.005).

**Table 2:** Distribution of pulmonary and bloodstream pathogens.

Variables^a^	Short-course therapy (*n* = 1355)	Long-course therapy (*n* = 1235)	p-value
MDR pathogen in lung	205 (15.1)	179 (14.5)	0.65
Staphylococcus in lung	209 (15.4)	263 (21.3)	<0.001
*E. coli* in lung	51 (3.8)	98 (7.9)	<0.001
K. pneumoniae in lung	53 (3.9)	81 (6.6)	0.002
P. aeruginosa in lung	95 (7.0)	110 (8.9)	0.074
S. maltophilia in lung	31 (2.3)	53 (4.3)	0.004
Accompanied by fungi in lung	470 (34.7)	587 (47.5)	<0.001
Accompanied by virus in lung	65 (4.8)	92 (7.4)	0.005
Gram-positive bacteria in blood	82 (6.1)	123 (10.0)	<0.001
Gram-negative bacteria in blood	33 (2.4)	39 (3.2)	0.26

MDR, Multidrug-Resistant; *E. coli, E. coli*; K. pneumoniae, *Klebsiella pneumoniae*; P. aeruginosa, *Pseudomonas aeruginosa*; S. maltophilia, *Stenotrophomonas maltophilia*.

“in lung” refers to pathogens isolated from respiratory specimens (e.g., endotracheal aspirate, sputum, or bronchoalveolar lavage). “in blood” refers to pathogens isolated from positive blood cultures. MDR pathogens were defined according to international criteria as non-susceptibility to at least one agent in three or more antimicrobial categories.

Categorical variables were expressed as n (%).

### Primary outcome

**Fig. 2:**
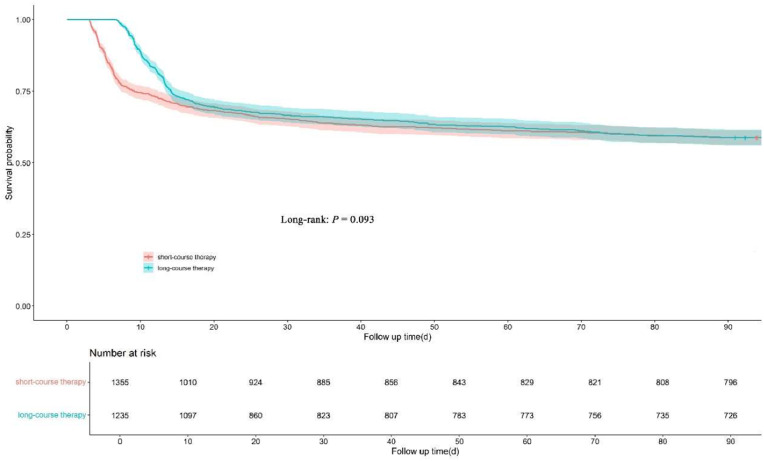
Kaplan-Meier survival curves for 90-day mortality in patients receiving short-course versus long-course antibiotic therapy.

There was no statistically significant difference in 90-day all-cause mortality between patients receiving short-course and long-course antibiotic therapy (41.3% vs. 41.2%, *p* = 0.98). Similarly, no differences were observed in 60-day mortality or in-hospital mortality (Table 3). Kaplan-Meier survival curves showed no significant difference in survival over time between the two groups (log-rank *p* = 0.093) (Fig. 2). After Propensity Score Matching (PSM) and balancing basic variables (SMD < 0.1), the outcome remained stable (Fig. S1, Fig. S2, Table S1, Table S2).

**Table 3:** Clinical outcomes of patients receiving short-course versus long-course antibiotic therapy.

Variables^a^	Short-course therapy (*n* = 1355)	Long-course therapy (*n* = 1235)	p-value
**Primary Outcome**			
90-day mortality, n (%)	559 (41.3)	509 (41.2)	0.98
**Secondary Outcomes**			
30-day mortality, n (%)	470 (34.7)	412 (33.4)	0.48
60-day mortality, n (%)	526 (38.8)	462 (37.4)	0.46
In-hospital mortality, n (%)	417 (30.8)	372 (30.1)	0.72
ICU length of stay, median (IQR), days	5.9 (4.7, 7.1)	11.3 (9.4, 13.7)	<0.001
Hospital length of stay, median (IQR), days	11.0 (7.1, 17.1)	16.8 (13.0, 24.2)	<0.001
Duration of vasopressor use, median (IQR), days	1.4 (0.0, 3.6)	3.62 (0.3, 7.8)	<0.001
Duration of mechanical ventilation, median (IQR), days	3.7 (2.7, 4.9)	7.0 (4.4, 9.4)	<0.001

Data are presented as number (%) or median (interquartile range) as appropriate.

ICU, Intensive Care Unit; IQR, Interquartile Range.

### Subgroup analyses

**Fig. 3:**
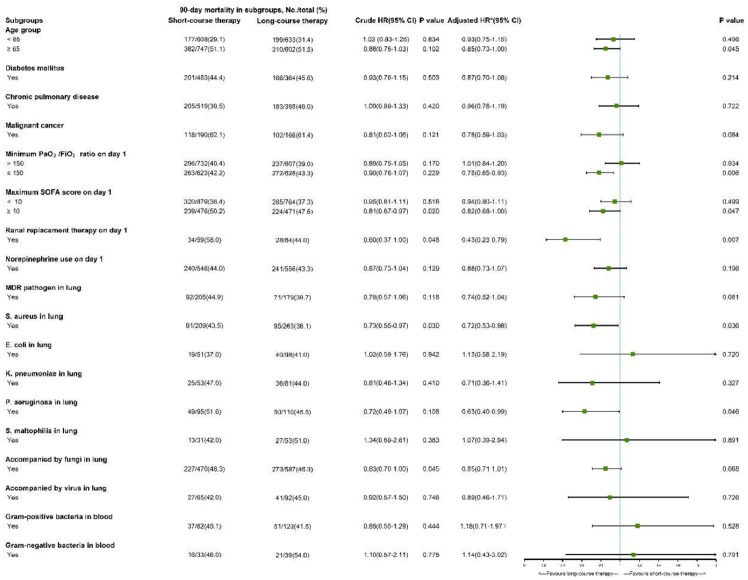
Subgroup analysis of mortality associated with sepsis patients with pneumonia and mechanical ventilation. All variables refer to presence at ICU admission or within the first 24 h unless otherwise specified. PaO_2_/FiO_2_: arterial oxygen partial pressure to fractional inspired oxygen ratio. SOFA, Sequential Organ Failure Assessment; MDR, multidrug-resistant; *S. aureus, Staphylococcus aureus; E. coli, Escherichia coli*; etc.

Univariate subgroup analyses demonstrated that the association between antibiotic duration and 90-day mortality was generally consistent across multiple strata, including age (< 65 vs. ≥ 65 years), diabetes mellitus, chronic pulmonary disease, malignancy, PaO_2_/FiO_2_ ratio on ICU day-1, vasopressor use, and microbiological pathogen groups. However, significant heterogeneity was observed in specific subgroups (Fig. 3).

In multivariable Cox regression models adjusted for all baseline covariates listed in Table 1, short-course therapy remained non-inferior in patients aged < 65-years (Hazard Ratio [HR = 0.93]; 95% Confidence Interval ‒ [95% CI 0.75–1.15]), those with diabetes (HR = 0.87; 95% CI 0.70–1.08), and those with maximum SOFA scores < 10 (HR = 0.94; 95% CI 0.80–1.11) (Fig. 3).

Conversely, in patients aged ≥ 65-years, long-course therapy was associated with a modest survival benefit (HR = 0.85; 95% CI 0.73–1.00; *p* = 0.045). Similar associations favoring long-course treatment were found in subgroups with minimum PaO_2_/FiO_2_ ≤ 150 (HR = 0.78; 95% CI 0.65–0.93), maximum SOFA score ≥ 10 (HR = 0.82; 95% CI 0.68–1.00), and those requiring renal replacement therapy on ICU day-1 (HR = 0.43; 95% CI 0.23–0.79) (Fig. 3).

Additionally, among patients with confirmed pulmonary *S. aureus* infection (HR = 0.72; 95% CI 0.53–0.98) or *Pseudomonas aeruginosa* infection (HR = 0.63; 95% CI 0.40–0.99), long-course antibiotic therapy was independently associated with reduced 90-day mortality (Fig. 3).

## Discussion

This study evaluated the clinical efficacy of short-course versus long-course antibiotic therapy in mechanically ventilated patients with sepsis secondary to pneumonia. The present findings demonstrate that short-course therapy was broadly non-inferior to long-course regimens in terms of 90-day all-cause mortality. However, extended therapy was associated with improved outcomes in specific high-risk subgroups, including older adults, patients with severe hypoxemia, those requiring renal replacement therapy, and those infected with *Staphylococcus aureus* or *Pseudomonas aeruginosa*. Given the observational design and residual confounding by indication, particularly the non-random, clinician-driven decision to shorten or extend antibiotics based on unmeasured clinical evolution, these subgroup findings are hypothesis‑generating and should not be interpreted as definitive evidence to change practice without randomized confirmation.

The comparable overall survival between the two groups reinforces the growing body of literature advocating for shorter antibiotic courses in critically ill patients with respiratory infections. Prior studies in Ventilator-Associated Pneumonia (VAP) and Hospital-Acquired Pneumonia (HAP) have shown that shorter durations (7–8 days) are as effective as longer regimens in most patients.^12^^,^^13^ The 2016 ATS/IDSA guidelines recommend a 7-day course for HAP/VAP in the absence of complications^14^, and meta-analyses have similarly supported this threshold.^15^^,^^16^ These results extend this evidence to a broader septic population requiring mechanical ventilation and support the view that abbreviated regimens, when appropriately selected, do not compromise survival.

Importantly, the subgroup analyses revealed that the protective effect of short-course therapy was not uniform across all patient strata. In older patients (≥ 65-years), those with severe hypoxemia (PaO_2_/FiO_2_.≤ 150), high illness severity (SOFA ≥ 10), and infections with virulent or difficult-to-treat organisms such as *S. aureus* and *P. aeruginosa*, extended antibiotic therapy was associated with better outcomes. Notably, Kaplan–Meier curves showed an early separation favoring longer therapy, although this did not persist as a definitive adjusted survival benefit and may reflect residual confounding. These findings are biologically plausible. However, it is important to recognize that the subgroups showing benefit from longer therapy (age ≥ 65, PaO_2_/FiO_2_ ≤ 150, RRT, *S. aureus, P. aeruginosa*) are underpowered and subject to multiple testing without correction. Therefore, the authors emphasize that these are exploratory, hypothesis-generating findings. Older adults often have reduced physiologic reserves and immunosenescence, potentially requiring longer antimicrobial coverage.^17^^,^^18^ Severe hypoxemia and organ dysfunction may reflect ongoing infection or impaired host clearance, warranting prolonged therapy.^19^ Moreover, infections caused by *P. aeruginosa* and *S. aureus* are known for their biofilm formation, resistance patterns, and propensity for relapse, often justifying extended regimens.^20^^,^^21^ The present data highlight the importance of tailoring antibiotic duration based on host and pathogen factors, rather than applying uniform treatment lengths to all sepsis patients.

Compared with prior literature, the present study is one of the few to specifically examine treatment duration in a large, homogenous cohort of ventilated pneumonia patients meeting Sepsis-3 criteria. Most existing trials have excluded patients with respiratory failure or have not stratified by sepsis status.^22^^,^^23^ Furthermore, the use of real-world, granular data from MIMIC-IV enabled robust adjustment for illness severity and pathogen profile, which enhances the external validity of the present findings. Although previous retrospective analyses have questioned the necessity of prolonged antibiotic exposure^24^, the present study offers additional nuance by identifying vulnerable subgroups where extended treatment may confer benefit.

Clinically, these findings could have implications for antimicrobial stewardship. Reducing unnecessary antibiotic duration is central to combating antimicrobial resistance, lowering costs, and minimizing adverse events such as *Clostridioides difficile* infection or nephrotoxicity.^25^^,^^26^ The present data support adopting short-course regimens in appropriately selected patients, consistent with stewardship principles and guideline recommendations. Simultaneously, the identification of subpopulations that may benefit from longer courses underscores the need for individualized clinical assessment rather than rigid adherence to standardized durations. In this context, dynamic monitoring of biomarkers (e.g., procalcitonin), physiological trends, and culture data could assist in optimizing therapy length.^27^

Nevertheless, several limitations warrant discussion. First, the retrospective design introduces the possibility of unmeasured confounding, particularly regarding clinical decision-making around antibiotic duration. Although the authors adjusted for multiple covariates, including SOFA score and vasopressor use, they could not account for treatment response, radiographic evolution, or clinician judgment, which may have influenced therapy duration. Second, microbiological data were limited to culture results; molecular diagnostics or antibiotic susceptibility profiles were not available. Third, the authors acknowledge that the lack of data on recurrent infections or relapse significantly limits understanding of the implications of antibiotic duration. The primary outcome of all-cause mortality cannot distinguish death from infection relapse versus other causes, which is a major gap for antibiotic duration studies. Without this relapse data, the safety of short-course therapy regarding infection recurrence cannot be adequately assessed. Fourth, therapy duration was categorized based on total consecutive days of systemic antibacterial administration, and transitions between agents or routes were not fully captured; specifically, oral step‑down therapy after initial intravenous treatment, interruptions in therapy (including gaps > 48 h), and changes in antibiotic class or combination regimens could not be consistently identified, potentially misclassifying some exposures and biasing the authors’ estimates toward the null. Finally, as MIMIC-IV is derived from a single academic center in the U.S., the authors recognize that antibiotic prescribing practices, local resistance patterns, and ICU protocols may differ elsewhere. Therefore, generalizability to other healthcare systems should be approached with caution, highlighting the need for further studies in diverse settings to enhance external validity.

In conclusion, this study provides evidence that in most mechanically ventilated patients with sepsis from pneumonia, short-course antibiotic therapy is as effective as longer regimens for 90-day survival. However, patients with higher acuity or high-risk pathogens may benefit from extended treatment. These findings support guideline-based stewardship efforts to shorten therapy where feasible, while advocating for individualized duration decisions based on host, pathogen, and disease severity. Future prospective trials should validate optimal antibiotic durations in well-defined septic subgroups and incorporate emerging tools such as biomarker-guided discontinuation and rapid microbiological diagnostics to refine treatment algorithms.

## Data availability statement

Publicly available datasets were analyzed in this study. This data can be found at https://mimic.physionet.org.

## Ethics approval and consent to participate

The use of MIMIC-IV data was approved under the database’s institutional review board protocol; data are fully de-identified and patient consent was waived per policy.

## Authors’ contributions

BW and ZYZ conceived and designed the study, collected and analyzed the data, conducted literature retrieval, and drafted the manuscript. BW, SQG, JM and ZYZ critically revised the final version of the manuscript. All authors ensured data integrity and accuracy.

## Funding

This work was supported by grants from the Sanming Project of Medicine in Shenzhen (SZSM202211016), Shenzhen Fund for Guangdong Provincial High-level Clinical Key Specialties (n° SZGSP006), and the 2025 project approved by the Shenzhen Health Economics Society (n° 202546; 2025270).

## Declaration of competing interest

The authors declare that the research was conducted in the absence of any commercial or financial relationships that could be construed as a potential conflict of interest.

## Appendix Supplementary materials

Image, application 1
