# Supplementary material for: Short- versus long-course antibiotic therapy in mechanically ventilated sepsis patients with pneumonia: a real-world cohort analysis
Source: Clinics (Sao Paulo). 2026 Apr 11;81:100932. doi: 10.1016/j.clinsp.2026.100932 (PMC13092029; doi:10.1016/j.clinsp.2026.100932)

**CLINICS-D-25-01495**

**Supplementary Material**

**Table S1** Baseline characteristics and clinical variables after propensity score matching.

| **Variables^a^** | **Short-course therapy (n = 1050)** | **Long-course therapy (n = 1050)** | **p-value** |
| --- | --- | --- | --- |
| Age (years) | 65.3 (54.3, 75.8) | 65.8 (54.1, 75.8) | 0.97 |
| Gender (male) | 608 (57.9) | 611 (58.2) | 0.89 |
| Weight (kg) | 78.9 (65.0, 95.2) | 79.6 (66.9, 97.1) | 0.30 |
| Emergency admission | 562 (53.5) | 562 (53.5) | 1.00 |
| White race | 646 (61.5) | 641 (61.0) | 0.82 |
| Medicare insurance | 582 (55.4) | 579 (55.1) | 0.90 |
| History of disease |  |  |  |
| Hypertension | 677 (64.5) | 674 (64.2) | 0.89 |
| Myocardial infarct | 192 (18.3) | 185 (17.6) | 0.69 |
| Diabetes Mellitus | 315 (30.0) | 325 (31.0) | 0.64 |
| Chronic pulmonary disease | 352 (33.5) | 357 (34.0) | 0.82 |
| Chronic kidney disease | 247 (23.5) | 243 (23.1) | 0.84 |
| Malignant cancer | 146 (13.9) | 144 (13.7) | 0.90 |
| Vital signs on day 1 |  |  |  |
| Maximum heart rate (bpm) | 110.0 (95.0, 126.0) | 112.0 (97.0, 126.0) | 0.43 |
| Maximum body temperature (°C) | 37.67 (37.17, 38.39) | 37.72 (37.22, 38.39) | 0.20 |
| Minimum PaO_2_/FiO_2_ ratio | 152.86 (102.86, 230.0) | 154.0 (95.0, 224.0) | 0.38 |
| Minimum MAP (mmHg) | 56.0 (48.0, 62.0) | 56.0 (49.0, 62.0) | 0.83 |
| Scoring system on day-1 |  |  |  |
| Maximum OASIS score | 39.0 (34.0, 44.0) | 40.0 (34.0, 45.0) | 0.12 |
| Maximum APS III score | 59.0 (45.0, 76.0) | 59.0 (46.0, 75.0) | 0.52 |
| Maximum SOFA score | 8.0 (5.0, 11.0) | 8.0 (6.0, 11.0) | 0.38 |
| Renal replacement therapy on day-1 | 53 (5.0) | 52 (5.0) | 0.92 |
| Norepinephrine use on day-1 | 457 (43.5) | 456 (43.4) | 0.96 |
| Time to antibiotic initiation (hours) | 4.0 (2.0, 11.38) | 3.38 (1.75, 9.38) | 0.022 |
| Time from admission to sepsis (hours) | 1.38 (0.75, 3.12) | 1.38 (0.75, 3.38) | 0.69 |

bpm, Beat per minute or breaths per minute; MAP, Mean Blood Pressure; OASIS, Oxford Acute Severity of Illness; SAP III, Simplified Acute Physiology score III; SOFA, Sequential Organ Failure Assessment.

^a^ Because all continuous variables were not normally distributed, the continuous variables were expressed as median (IQR). Categorical variables were expressed as n (%).

**Table S2** Clinical outcomes of patients receiving short-course versus long-course antibiotic therapy after Propensity Score Matching.

| **Variables^a^** | **Short-course therapy (n=1050)** | **Long-course therapy (n=1050)** | **p-value** |
| --- | --- | --- | --- |
| **Primary Outcome** |  |  |  |
| 90-day mortality, n (%) | 431 (41.0) | 424 (40.4) | 0.76 |
| **Secondary Outcomes** |  |  |  |
| 30-day mortality, n (%) | 366 (34.9) | 349 (33.2) | 0.43 |
| 60-day mortality, n (%) | 406 (38.7) | 389 (37.0) | 0.44 |
| In-hospital mortality, n (%) | 326 (31.0) | 317 (30.2) |  |
| ICU length of stay, median (IQR), days | 6.0 (4.7, 7.1) | 11.3 (9.3, 13.7) | <0.001 |
| Hospital length of stay, median (IQR), days | 11.0 (7.2, 17.3) | 16.7 (12.9, 23.7) | <0.001 |
| Duration of vasopressor use, median (IQR), days | 1.5 (0.0, 3.7) | 3.4 (0.1, 7.8) | <0.001 |
| Duration of mechanical ventilation, median (IQR), days | 3.8 (2.8, 4.9) | 7.0 (4.4, 9.5) | <0.001 |

Data are presented as number (%) or median (interquartile range) as appropriate.

ICU, Intensive Care Unit; IQR, Interquartile Range.

**Figure S1** Before and after propensity score matching the difference of baseline characteristics between the two groups.


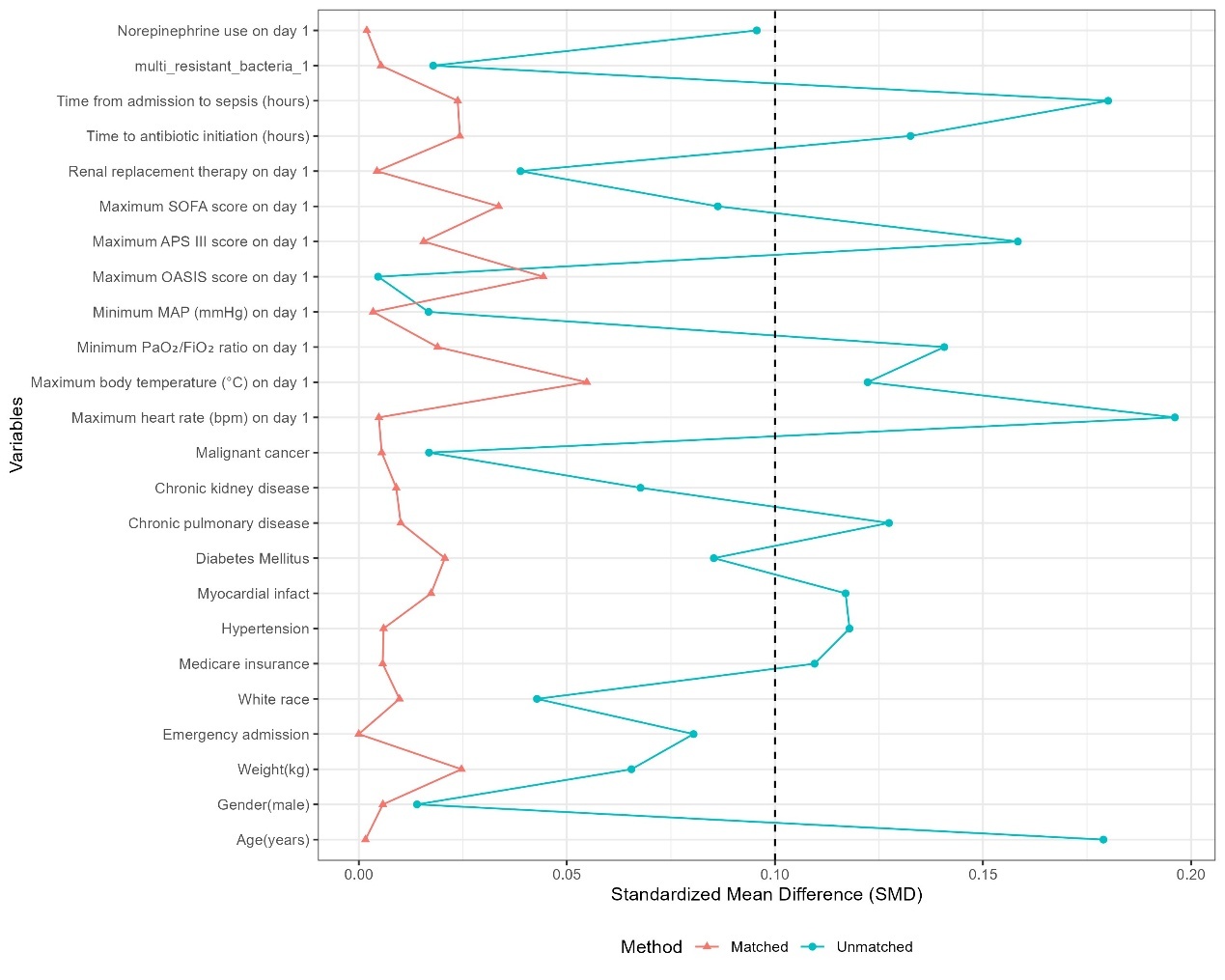


**Figure S2** Kaplan-Meier survival curves for 90-day mortality in patients receiving short-course versus long-course antibiotic therapy after Propensity Score Matching.


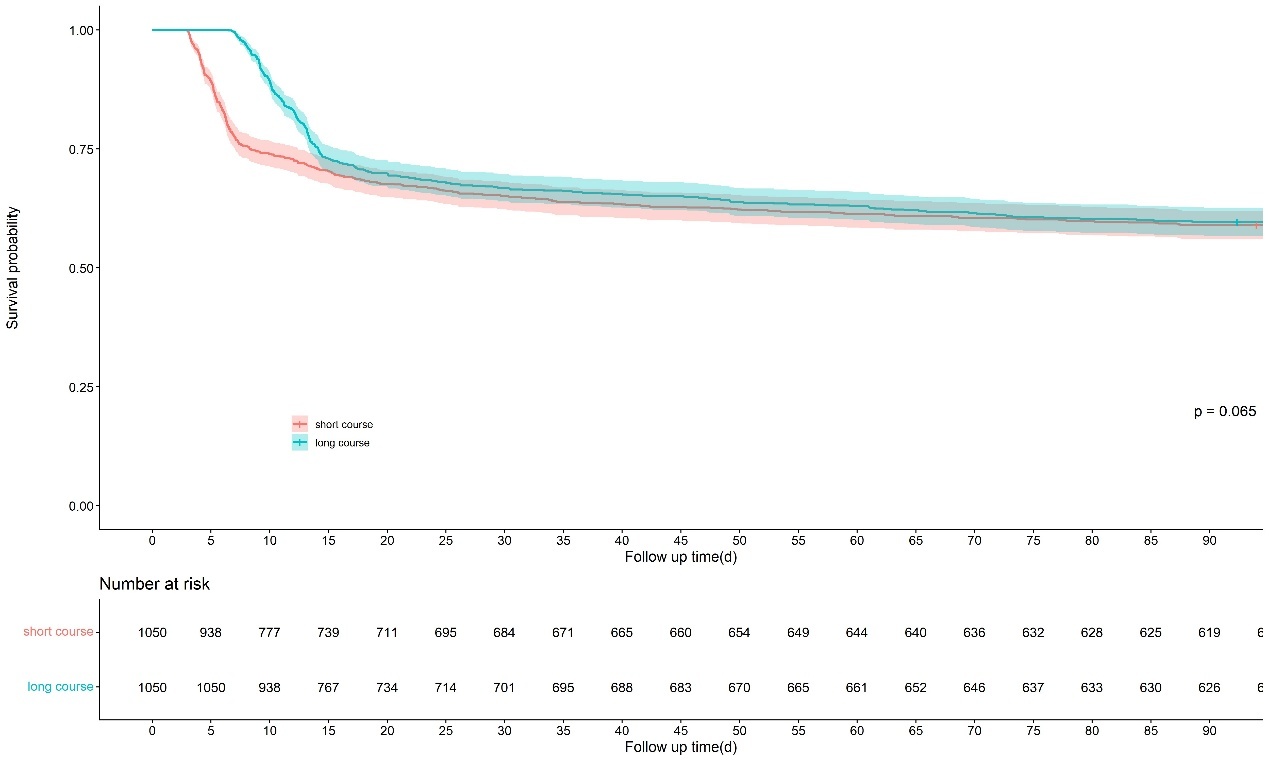

Supplement: Supplementary file 1 [file mmc1.docx]
